# Predictive Biomarkers for Postmyocardial Infarction Heart Failure Using Machine Learning: A Secondary Analysis of a Cohort Study

**DOI:** 10.1155/2021/2903543

**Published:** 2021-12-13

**Authors:** Feng Li, Jin-Yu Sun, Li-Da Wu, Qiang Qu, Zhen-Ye Zhang, Xu-Fei Chen, Jun-Yan Kan, Chao Wang, Ru-Xing Wang

**Affiliations:** ^1^Department of Cardiology, Wuxi People's Hospital, Nanjing Medical University, Wuxi 214023, China; ^2^Department of Cardiology, The First Affiliated Hospital of Nanjing Medical University, Nanjing 210029, China; ^3^Department of Cardiology, The First Affiliated Hospital of Guangxi Medical University, Nanning 530021, China

## Abstract

**Background:**

There are few biomarkers with an excellent predictive value for postacute myocardial infarction (MI) patients who developed heart failure (HF). This study aimed to screen candidate biomarkers to predict post-MI HF.

**Methods:**

This is a secondary analysis of a single-center cohort study including nine post-MI HF patients and eight post-MI patients who remained HF-free over a 6-month follow-up. Transcriptional profiling was analyzed using the whole blood samples collected at admission, discharge, and 1-month follow-up. We screened differentially expressed genes and identified key modules using weighted gene coexpression network analysis. We confirmed the candidate biomarkers using the developed external datasets on post-MI HF. The receiver operating characteristic curves were created to evaluate the predictive value of these candidate biomarkers.

**Results:**

A total of 6,778, 1,136, and 1,974 genes (dataset 1) were differently expressed at admission, discharge, and 1-month follow-up, respectively. The white and royal blue modules were most significantly correlated with post-MI HF (dataset 2). After overlapping dataset 1, dataset 2, and external datasets (dataset 3), we identified five candidate biomarkers, including *FCGR2A*, *GSDMB*, *MIR330*, *MED1*, and *SQSTM1*. When *GSDMB* and *SQSTM1* were combined, the area under the curve achieved 1.00, 0.85, and 0.89 in admission, discharge, and 1-month follow-up, respectively.

**Conclusions:**

This study demonstrates that *FCGR2A*, *GSDMB*, *MIR330*, *MED1*, and *SQSTM1* are the candidate predictive biomarker genes for post-MI HF, and the combination of *GSDMB* and *SQSTM1* has a high predictive value.

## 1. Introduction

Heart failure (HF) is one of the primary long-term complications of acute myocardial infarction (MI). Meanwhile, post-MI HF has been identified as a time-dependent variable significantly related to mortality with a hazard ratio of 3.31 [1–3]. Screening the post-MI HF genes served as novel candidate biomarkers facilitates exactly diagnosis and timely intervention. However, despite many proposed biomarkers involving post-MI HF, few of them have gained widespread acceptance and application in clinical practice [4].

We analyzed the gene expression profile of post-MI HF patients and those who remained HF-free over a 6-month follow-up using plasma samples collected at admission, discharge, and 1-month follow-up. Differential expression analysis and weighted gene coexpression network analysis (WGCNA) were combined to screen the top-ranked circulating candidates. In addition, we performed enrichment analysis to illustrate the potential influence on progression from MI to HF using functional annotation algorithms. Moreover, we confirmed the differentially expressed genes (DEGs) and key modules using external datasets from 2 different acute MI patient cohorts, 4 single-cardiac cell transcriptomic studies [5], and 12 ischemic cardiomyopathy patients' expression profiles [6]. This study aimed to identify circulating biomarkers to predict post-MI HF using machine learning methods.

## 2. Methods

### 2.1. Data Acquisition

**Figure 1:**
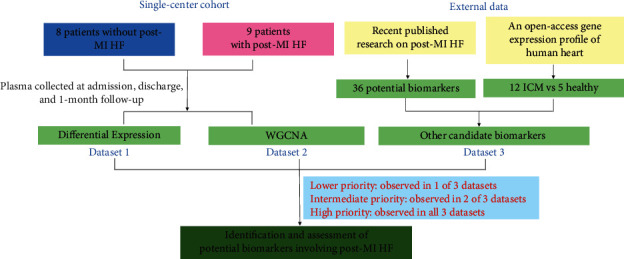
Study workflow diagram summarizing the entire study design. MI: myocardial infarction; HF: heart failure; ICM: ischemic cardiomyopathy; WGCNA: weighted gene coexpression network analysis.

A total of 64 samples from patients with ST-elevation MI were enrolled from the First Chair and Department of Cardiology of the Medical University of Warsaw, with the approval of the Ethics Committee of the Ain Shams the Faculty of Medicine [7]. All 17 patients were indicated for direct percutaneous coronary intervention. Coronary angiography, angioplasty of the infarct-related artery, and pharmacological therapy were performed following the 2008 European Society of Cardiology guidelines for acute myocardial infarction [8]. Whole blood samples were collected at the time point of admission (first day of MI), discharge (4 to 6 days after MI), and 1-month follow-up, respectively. According to the manufacturer's instructions, the transcriptional profiling was analyzed using Human Gene 1.0 ST Array (Affymetrix, Santa Clara, CA, USA; Platform GPL6244). The involvement of this study did not influence treatment. All participants were provided written informed consent following the Declaration of Helsinki. This study was a secondary data analysis on publicly available data, and the raw data were acquired from the Gene Expression Omnibus database (https://www.ncbi.nlm.nih.gov/geo/) [7].  Figure 1 shows a flow diagram summarizing the entire study design.

### 2.2. Data Processing and Probe Reannotation

Log_2_-transformation, background correction, and quantile normalization were performed on the raw gene expression profiles using the linear models for the microarray data (limma) algorithm. Then, the probe serial numbers were converted into gene symbols according to the annotation file provided by the manufacturer. When a single gene was mapped by more than one probe, the average expression level of this gene was calculated. Finally, the expression profile containing 23,307 genes was further processed.

### 2.3. Clustering Analysis and Visualization

Clustering analysis is a powerful tool to perform molecular classification among samples and identify subtype characterization [9–11]. Among the many clustering algorithms, hierarchical cluster analysis and k-means clustering are the two prominent representatives, whereas t-distributed stochastic neighbor embedding analysis and principal component analysis are widely used unsupervised methods to reduce dimensions of expression data.

The processed expression data were first analyzed by the k-means cluster method and visualized using a heatmap. Then, we performed an unsupervised hierarchical cluster analysis with a scale-free network and topological overlaps. Meanwhile, hierarchical cluster analysis is a cluster analysis method to create a hierarchy of clusters and thus group patients with similar gene expressions into the same clusters [12,13]. Additionally, we ran the discriminant analysis using t-distributed stochastic neighbor embedding analysis, a nonlinear dimensionality reduction algorithm well-suited for visualizing high-dimensional data [14,15]. In this study, hierarchical cluster and t-distributed stochastic neighbor embedding analysis were performed on the full set (all the four time points) of detected genes, which aimed to illustrate the general difference in expression patterner between the post-MI HF and non-HF groups.

Moreover, we performed principal component analysis on the expression data of admission, discharge, and 1-month follow-up, respectively. The principal component analysis is a widely used distance-based statistical algorithm that reduces the dimensionality of complex datasets, increases interpretability, and minimizes information loss [14–19]. An appropriate time point with good distinguishing ability will be selected based on the expression parameter revealed by principal component analysis.

### 2.4. Screening Differentially Expressed Genes (DEGs)

Fold change is a univariate filter method to compare the absolute expression value change between two groups, and it has been widely used as a threshold for screening possible biomarkers. We analyzed the gene expression profile acquired at three time points (admission, discharge, and 1-month follow-up) and screened DEGs between the post-MI HF and non-HF groups based on log_2_ fold change expression using the limma method [20]. We assumed that the difference in blood samples might be smaller compared with tissue samples (like heart tissue). Therefore, to avoid eliminating excessive candidate biomarkers, we set a lower threshold of fold change >1.1 and *P* value < 0.05. The DEGs were visualized as a volcano plot and heatmap using the “ggplot2” and “pheatmap” package in R.

### 2.5. Construction of WGCNA

WGCNA is a bioinformatics algorithm to explore the transcriptome expression patterns across genes, identify gene modules associated with complex disease features, and reveal the biologically functional interpretations of network modules [21–24]. Based on the time-series gene expression profiles, we used the one-step network construction function of the “WGCNA” package (version 1.60) for constructing the coexpression network and identifying key modules. Scale independence and mean connectivity were calculated using a gradient method with a range of 1 to 20, and the power value was selected with a threshold of independence degree >0.8. The minimal module size and the merge cut height were set as 30 and 0.3, respectively. After module construction, we summarized the module eigengene according to the first module principal component to evaluate the significance of each module, and the module-trait relationships were assessed based on the correlation between module eigengenes and clinical traits. Furthermore, we calculated all genes' average absolute gene significance within one module and evaluated the correlation strength accordingly. In addition, the gene significance value was defined by log10-transformed *P* value in the linear regression between expression and clinical traits. The modules with the highest MS values were considered as the key modules [21].

After constructing coexpression networks, we further evaluated the preservation levels of key modules using module preservation analysis, which summarizes different preservation statistics into one single overall measure of preservation (i.e., Zsummary value). Zsummary is a statistic value composed of multiple statistics related to density connectivity [25]. Generally, a higher Zsummary value suggested the more substantial evidence that a module should be preserved: Zsummary value less than 2 indicated “no evidence,” Zsummary value between 2 and 10 indicated “weak evidence,” and Zsummary value higher than 10 indicated strong evidence. However, the Zsummary value tends to increase with the rise of module size, and therefore, it is unappropriated to use the Zsummary value to perform preservation analysis on modules with distinct sizes. In that case, medianRank, which is calculated based on the observed preservation statistics and not affected by module size, should also be applied [26]. A module with a lower medianRank value is more preserved than those with a higher medianRank.

### 2.6. Enrichment Analysis and Protein-Protein Interaction (PPI) Network

To reveal the roles of key modules in the progression of post-MI HF, we ran gene ontology (GO) enrichment analysis using the “clusterProfiler” package. Moreover, we performed association and enrichment analysis based on DisGeNET [27] database and visualized using Metascape, which was a tool to systematically analyze and interpret OMICs-based research [28]. DisGeNET, a gene-disease associations database, contains publicly available collections of genes and human disease-associated variants [27]. In addition, we ran a PPI enrichment analysis on genes from key modules and created an interaction network. The molecular complex detection algorithm was also applied to detect densely connected network components [29].

### 2.7. Identification of Potential Biomarkers and Expression Analysis

To identify potential biomarkers for post-MI HF, DEGs (dataset 1) were cross-referenced with genes from key modules (dataset 2). We considered the biomarkers would be reproducible if they were identified by both expression analysis and coexpression network analysis at the same time. In parallel, we included other candidate biomarkers from external datasets (dataset 3): (1) a recently published research combining aptamer-based proteomics from 2 different acute MI patient cohorts and 4 single-cardiac cell transcriptomic studies, which identified 36 potential circulating biomarkers [5]; (2) an open-access gene expression profile of human heart evaluating the influence of heart failure on human nucleocytoplasmic transport-related genes: 12 samples from ischemic cardiomyopathy and 5 samples from control hearts [6]. Then, we ranked biomarkers according to 3 priorities: lower priority (observed in 1 of 3 datasets), intermediate priority (observed in 2 of 3 datasets), and high priority (observed in all three datasets).

### 2.8. Statistical Analysis

Continuous variables were represented as mean ± standard deviation (normal distribution) or median + interquartile range (skewed distribution). Categorical variables were presented as percentages. The one-way ANOVA test, Kruskal–Wallis test, and chi-square test were used to determine statistical differences, as appropriate. The receiver operating characteristic (ROC) curves were created, and the area under the curve (AUC) was calculated to assess the predictive value of these possible biomarkers. All statistical analysis was performed by R software version 3.6.1 (R Foundation for Statistical Computing, Vienna). *P* < 0.05 was considered as statistical significance.

## 3. Results

### 3.1. Clinical Characteristics of the Study Population

This study included 17 patients with myocardial infarction who volunteered for a six-month visit. All these patients were diagnosed with STEMI and received coronary angiography, angioplasty, and pharmacological treatment following current guidelines [8]. After six months, 9 patients were diagnosed with HF (post-MI HF group), and the other 8 individuals were grouped into the post-MI non-HF group. No significant difference was observed in age, sex, body mass index, hypertension, diabetes, smoking, hypercholesterolemia, anterior myocardial infarction, and medications (beta-blockers, aspirin, clopidogrel, statins, and angiotensin-converting enzyme inhibitors) at baseline (all *P* > 0.05). However, the post-MI HF group showed higher NT-proBNP (918.3 ± 848.5 *vs.* 62 ± 14.1 pg/mL, *P* < 0.001), lower LVEF (39.3 ± 8.4 *vs.* 66.8 ± 1.9%, *P* = 0.001), and more administration of diuretics (7 *vs.* 1, *P* = 0.015) compared with the non-HF group. Baseline demographic and clinical characteristics have been summarized in the parent study [7].

### 3.2. Clustering Analysis and Visualization

**Figure 2:**
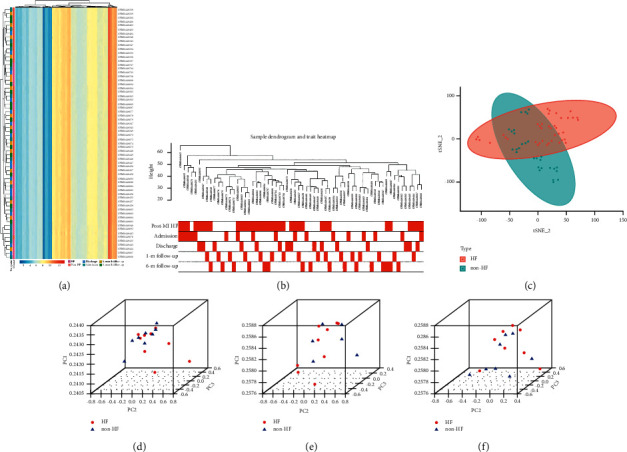
Clustering analysis of the expression profile. (a) K-means clustering, (b) hierarchical cluster, and (c) t-distributed stochastic neighbor embedding analysis. Principal component analysis on the sample collected at (d) admission, (e) discharge, and (f) 1-month follow-up. HF: heart failure; PC: principal component.

K-means cluster analysis indicated a distinct expression patterner between the HF and non-HF groups, although only limited expression similarity was observed in samples from the same time point (Figure 2(a)). Consistently, hierarchical cluster and t-distributed stochastic neighbor embedding analysis suggested that post-MI HF patients showed a different expression patterner compared with non-HF patients (Figures 2(b) and 2(c)). Principal component analysis on the expression data of three time points showed that the expression at admission and discharge might be appropriate time points with a good distinguishing ability (Figures 2(d)–2(f)).

### 3.3. Differential Gene Expression Profiling in HF and Non-HF Groups

**Figure 3:**
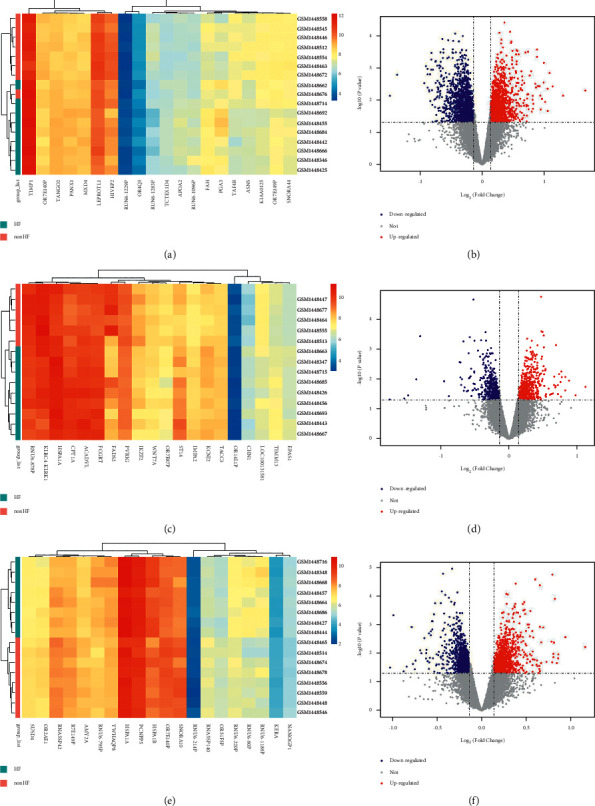
The expression heatmap and volcanic plots of the differential gene expression between post-MI HF and non-HF patients. The analysis was performed on the sample collected at (a, b) admission, (c, d) discharge, and (e, f) 1-month follow-up, respectively. MI: myocardial infarction; HF: heart failure.

For the expression data acquired at admission, 3,556 genes were significantly upregulated, whereas 3,222 genes were significantly downregulated (Figures 3(a) and 3(b)). At the time point of discharge, differential expression analysis identified a total of 1,136 genes associated with post-MI HF events (519 up- and 617 downregulated in HF patients; Figures 3(c) and 3(d)). However, 1,974 genes were differently expressed at 1 month (950 up- and 1024 downregulated in HF patients; Figures 3(e)and 3(f)).

### 3.4. Weighted Coexpression Network Construction and Key Modules Identification

**Figure 4:**
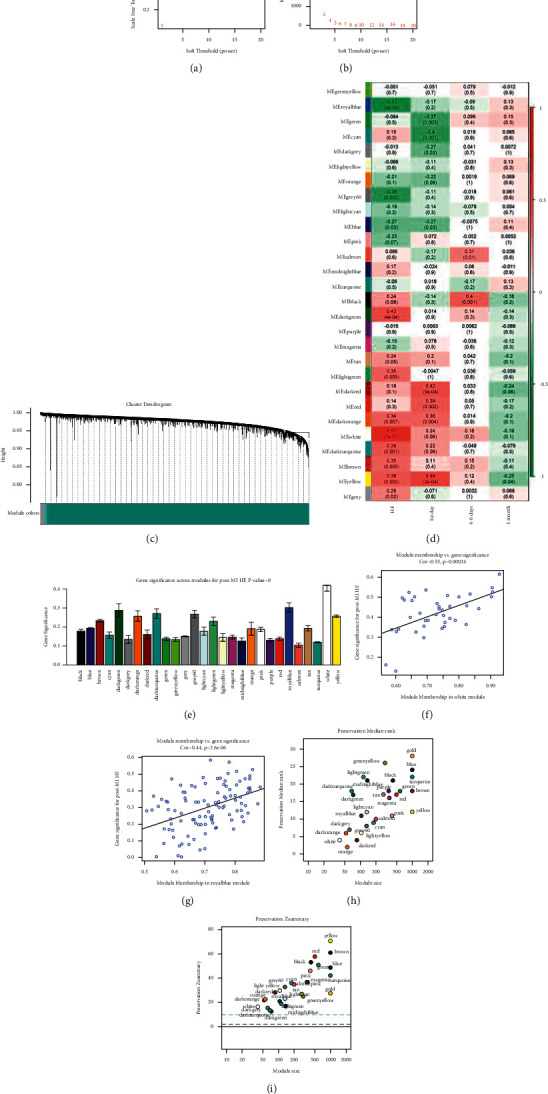
Network construction and analysis of the weighted coexpressed genes. (a) Analysis of the scale-free fit index and (b) the mean connectivity for various soft-thresholding powers. The soft-thresholding power of 8 was selected based on the scale-free topology criterion. (c) Dendrogram clustered using a dissimilarity measure (1-TOM). Each color in the dendrogram indicates one coexpression module, and every branch stands for a signal gene. (d) Heatmap of the correlation between module eigengenes and the disease status of post-MI HF. (e) Distribution of gene significance in the modules associated with post-MI HF. The white and royal blue modules were most significantly positively or negatively correlated with post-MI HF, respectively. Scatter plot of module eigengenes in the (f) white module and (g) royal blue module. (h) Module preservation analysis based on Zsummary. Each point represents a module, and the dashed blue and green lines indicate the threshold of 2 and 10, respectively. A module with Zsummary of <5 would be considered as nonpreserved. (i) MedianRank score analysis of different modules. MI: myocardial infarction; HF: heart failure.

The soft-thresholding power of 8 was selected according to the scale-free topology criterion (scale-free *R*^2^ = 0.81, Figures 4(a) and 4(b)), and 28 modules were created (Figure 4(c)). All the genes that could not be put into any other modules were included in the grey module, and the grey module was excluded from the following research. Next, we analyzed the association between modules and clinical traits, including the diagnosis of HF and follow-up time (Figures 4(d) and 4(e)). The white and royal blue modules were most significantly positively or negatively correlated with post-MI HF, respectively. Accordingly, white and royal blue modules were identified as the key modules. A total of 40 and 105 genes were included in the white and royal blue modules, respectively. In Figures 4(f) and 4(g), we illustrated the correlation between module membership and gene significance in white (correlation coefficient = 0.91, *P* < 1e-200) and royal blue module (correlation coefficient = 0.74, *P* = 1.8e-111).  Figure 4(h) shows the module preservation statistics, and the Z_summary_ values of both white and royal blue modules were more than 10. Additionally, Figure 4(i) illustrates the medianRank score analysis of different modules.

### 3.5. Enrichment Analysis of Key Modules and Interaction Network

**Figure 5:**
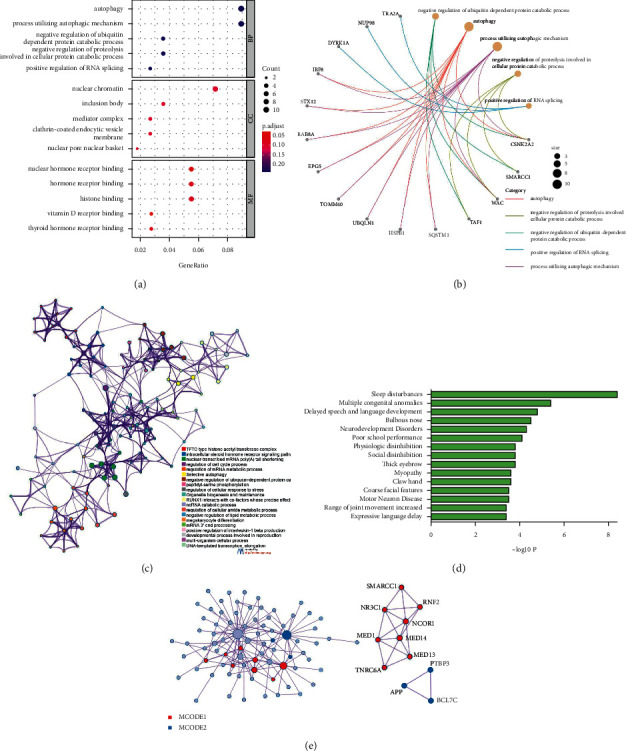
Enrichment analysis of key modules and interaction network. (a) GO analysis on white and royal blue modules. The significance of enrichment gradually increases from blue to red, and the size of the dots indicates the number of genes contained in the corresponding pathway. (b) Gene network of GO analysis. (c) The network of enriched terms. Each node represents an enriched term and is colored by cluster ID. Nodes sharing the same cluster ID are typically close to each other. (d) Summary of enrichment analysis in DisGeNET. (e) The PPI network of the genes in key modules. GO: Gene Ontology; PPI: protein-protein interaction.

We ran enrichment analysis on the key modules using the Gene Ontology database. As shown in Figure 5(a), enriched biological processes were mainly involved in autophagy, a process utilizing autophagic mechanism, negative regulation of ubiquitin-dependent protein catabolic process, negative regulation of proteolysis involved in cellular protein catabolic process, and positive regulation of RNA splicing. The cellular components were mainly enriched in nuclear chromatin, inclusion body, mediator complex, clathrin-coated endocytic vesicle membrane, and nuclear pore nuclear basket. Enriched molecular functions mainly involved nuclear hormone receptor binding, hormone receptor binding, histone binding, vitamin D receptor binding, and thyroid hormone receptor binding. Additionally, Figure 5(b) shows the gene network of GO analysis, and the network of enriched terms is shown in Figure 5(c). Moreover, the enrichment analysis in DisGeNET revealed that genes in the key modules were associated with sleep disturbances, multiple congenital anomalies, delayed speech and language development, bulbous nose, and neurodevelopmental disorders (Figure 5(d)). Furthermore, the PPI network was illustrated in Figure 5(e), and 2 cluster subnetworks (including *SMARCC1*, *NR3C1*, *RNF2*, *NCOR1*, *MED1*, *MED14*, *TNRC6A*, *APP*, *PTBP3*, and *BCL7C*) were created using the molecular complex detection algorithm.

### 3.6. Identification of Potential Biomarkers and Expression Analysis

**Figure 6:**
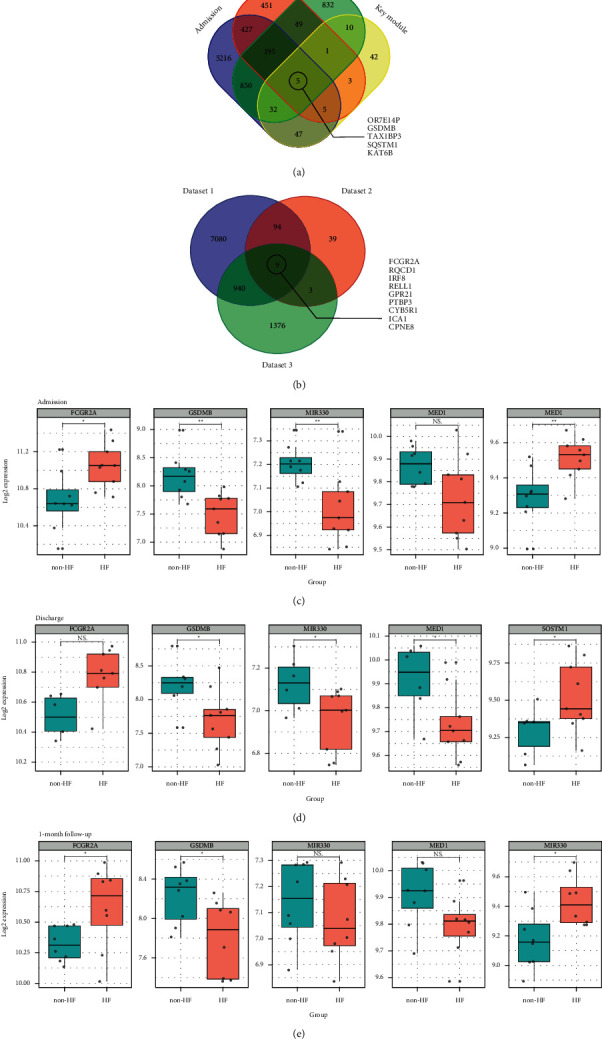
Identification of potential biomarkers and expression analysis for post-MI HF. (a) The Venn diagram of genes from the blue and yellow modules and DEGs from samples acquired at admission, discharge, and 1-month follow-up. (b) The Venn diagram of genes from dataset 1, dataset 2, and dataset 3. The expression levels of the 5 candidate genes in post-MI HF and non-HF patients at (c) admission, (d) discharge, and (e) 1-month follow-up. MI: myocardial infarction; HF: heart failure; DEGs: differentially expressed genes; NS: no significance. ^*∗*^*P* < 0.05; ^*∗∗*^*P* < 0.01; ^*∗∗∗*^*P* < 0.001.

Dataset 1 included 200 DEGs from 3 time points, and dataset 2 included 145 genes from key modules. After overlapping dataset 1 and dataset 2, a total of 5 genes were acquired, including *OR7E14P*, *GSDMB*, *TAX1BP3*, *SQSTM1,* and *KAT6B*. The detailed cross-reference information was provided in Figure 6(a). Moreover, 9 genes were found in all three datasets and considered high-priority candidates, including *FCGR2A*, *RQCD1*, *IRF8*, *RELL1*, *GPR21*, *PTBP3*, *CYB5R1*, *ICA1*, and *CPNE8* (Figure 6(b)). Based on a literature search, we identified 5 genes that might most effectively differentiate the post-MI HF patients from those without HF: *FCGR2A*, *GSDMB*, *MIR330*, *MED1*, and *SQSTM1*. Figures 6(c)–6(e) shows the expression level of these genes at admission, discharge, and 1 month after discharge, respectively.

### 3.7. Accuracy of Biomarkers for Predicting Post-MI HF

To evaluate the predictive value of the 5 biomarkers, we created ROC curves and calculated the AUC at all 3 time points, respectively (Table 1). When combining *GSDMB* and *SQSTM1*, the AUCs achieved 1.00, 0.85, and 0.89 in admission, discharge, and 1-month follow-up, respectively.

## 4. Discussions

In this study, we performed a secondary analysis of a cohort study using machine learning including nine post-MI HF patients and eight post-MI patients who remained HF-free over a 6-month follow-up. The main findings are as follows. (1) Five candidate biomarkers (including *FCGR2A*, *GSDMB*, *MIR330*, *MED1,* and *SQSTM1*) were identified, which might most effectively differentiate the post-MI HF patients from those without HF. (2) When combining *GSDMB* and *SQSTM1*, the AUC achieved as high as 1.00, 0.85, and 0.89 in admission, discharge, and 1-month follow-up, respectively, indicating a high predictive value for post-MI HF.

*FCGR2A*, also named Fc*γ*RIIa, is a low-affinity receptor for the constant fragment of immunoglobulin G, mainly expressed on platelets' surface. Calverley et al. [30] reported an increased level of *FCGR2A* in patients with myocardial infarction, unstable angina, and ischemic stroke. Schneider et al. [31] analyzed the expression level of *FCGR2A* in post-MI patients and found a 4-fold greater risk of subsequent MI, stroke, and death in those with higher platelet *FCGR2A* expression. In our study, we revealed that *FCGR2A* was significantly upregulated in post-MI HF patients. Engagement of *FCGR2A* on platelets by immune complexes will trigger intracellular signaling events and lead to platelet activation and aggregation. Multiple studies have revealed that HF was significantly associated with abnormal platelet morphology and function [32, 33]. In addition, HF patients have higher mean platelet volume [34], increased whole blood aggregation [35], and elevated platelet-derived adhesion molecules [36]. Potential mechanisms include hemodynamic and vascular factors, secretion of cytokines like C-C chemokines, and renin-angiotensin system activation [32].

Gasdermins (*GSDMs*) are a family of functionally diverse proteins expressed in various cell types and tissues, and *GSDMs* have been well demonstrated to be involved in pyroptosis, a proinflammatory type of regular cell death [37]. It has been reported that *GSDMB* promotes noncanonical pyroptosis by enhancing caspase-4 activity and *GSDMD* cleavage [38]. With the deepening understanding of HF and chronic inflammation, pyroptosis has been revealed as having an important role in HF [39]. The pyroptosis of myocardial cells leads to the irreversible loss of cardiomyocytes, whereas pyroptosis of cardiac fibroblasts results in myocardial fibrosis and cardiac hypertrophy, which leads to the adverse change in cardiac structure and function and will eventually result in HF. Moreover, accumulating studies revealed that sleep disturbances significantly increased cellular stress, inflammation, and myoblast pyroptosis, leading to the development of HF [40–42]. Interestingly, our results revealed that *GSDMB* was differently expressed in all three time points and included in key modules showing high similarity with sleep disturbances-associated genes, suggesting its important role in the development of post-MI HF.

*SQSTM1* (also known as p62), a multifunctional protein consisting of a series of domains, acts in concert with binding partners to regulate the cellular process, especially autophagy [43]. As an autophagy receptor, *SQSTM1* has been recognized as an autophagy marker [44]. Autophagy is a self-degradative process for delivering aggregating proteins and damaged organelles to lysosomes for degradation, protecting cells from intracellular stress, and providing essential energy for starving cells [45]. However, the exact mechanisms between autophagy and HF remain largely vague despite the many studies. Current evidence indicates the key role of autophagy in protecting myocardial cells against HF, while overactivation of autophagy will contribute to the progress of HF [46,47]. In the early stage of HF, activated autophagy increases protein degradation, reduces myocardial hypertrophy, and antagonizes ventricular hypertrophy. On the contrary, autophagy promotes cardiomyocyte death and accelerates the deteriorating progression of HF. In our study, the expression of *SQSTM1* was significantly increased in post-MI HF, which suggested that excessive autophagy with MI might contribute to the development of HF. In addition, our results showed that the combination of *GSDMB* and *SQSTM1* had a high predictive value for post-MI HF, indicating that pyroptosis and autophagy played a jointly promoting role in the development of post-MI HF.

Mediator, a multisubunit nuclear complex, is a major component of eukaryotic transcription machinery that served as a bridge between transcription factors and RNA polymerase II [48]. Studies have demonstrated that Med1 (a subunit of mediator) plays an important role in regulating vital cardiac gene expression and maintaining normal heart function. Reportedly, deletion of *Med1* may lead to cardiac function abnormalities, including left ventricular dilation, decreased ejection fraction, and pathological ventricular remodeling [49,50]. Hall et al. [51] revealed that deletion of *Med1* in cardiomyocytes deregulated more than 5000 genes and promoted the development of acute HF. Underlying mechanisms may be involved in the deregulated expression of genes in calcium signaling, cardiac muscle contraction, and mitochondrial metabolic functions, accompanied by the downregulated expression of *Med1* [52]. Interestingly, Bai et al. [53] had reported that *Med1* in macrophages has an antiatherosclerotic role by suppressing the expression of proinflammatory genes via PPAR*γ*-regulated transactivation [54, 55], suggesting its protective role in the cardiovascular system. Similarly, our study showed that *Med1* was significantly downregulated in patients with post-MI HF, indicating it may be served as an effective biomarker for HF.

MiRNAs are a class of small noncoding RNAs, which function as regulators of gene expression at the posttranscriptional level [56]. Ren et al. [57] demonstrated that overexpression of *MIR330* in acute coronary syndrome alleviated acute coronary syndrome by suppressing atherosclerotic plaque formation and enhancing vascular endothelial cell proliferation through the WNT signaling pathway. Moreover, Wei et al. [58] reported that upregulated *MIR330* might lead to stable carotid plaques by targeting Talin-1 in symptomatic carotid stenosis patients. However, in another research [59], overexpression of *MIR‐330* was reported to promote left ventricular remodeling, increase myocardial infarction sizes, and aggravate myocardial ischemia-reperfusion injury during coronary recanalization. Different downstream pathways exert distinct biological effects, and the role of *MIR330* in post-MI HF remains to be further studied.

## 5. Limitation

Several limitations should be highlighted in our study. First, our study belongs to a secondary analysis of a cohort study. In the parent study [7], the study group and validation group were created, and microarrays were used to identify a set of genes associated with post-MI development HF in the early phase of MI, especially on admission. Differently, in our study, we focused on the study group of the parent study and performed a more in-depth analysis of the whole blood samples collected at admission, discharge, and 1-month follow-up to screen candidate biomarkers to predict post-MI HF in the early stage using differential expression analysis and WGCNA. More importantly, the developed external datasets on post-MI HF were introduced to confirm the candidate biomarkers, which facilitated increasing sample sizes and improving the reliability of results. Second, the datasets contained different cardiac models and pathologies and failed to include a strictly post-MI HF disease [5, 6], which posed a risk of introducing false positives and false negatives. However, these pathologies shared many underlying features and were likely to exhibit similar biomarker profiles [5]. Moreover, despite included external datasets, our study still had relatively smaller sample sizes, which may have an effect on the stability of results, reduce the test efficiency, and cause possible bias to the research results; accordingly, external validation with a larger cohort is still required to demonstrate their reliability, and meanwhile, further studies should be required to elucidate the underlying mechanisms. In addition, our screening tools have their limitations; therefore, candidate biomarkers need further validation in clinical and experimental studies.

## 6. Conclusions

This study demonstrates that *FCGR2A*, *GSDMB*, *MIR330*, *MED1,* and *SQSTM1* are the candidate biomarkers for the progression of HF after MI, and the combination of *GSDMB* and *SQSTM1* has the highest predictive value. Following studies are required to further validate the predictive accuracy and clarify the underlying mechanisms.

## Figures and Tables

**Table 1:** The value of AUC for five candidate biomarkers of post-MI HF at all three time points.

	Admission	Discharge	1-month follow-up
*FCGR2A*	0.8472	0.8333	0.7969
*GSDMB*	0.9028	0.8148	0.8125
*MIR330*	0.8750	0.7963	0.6406
*MED1*	0.7361	0.8333	0.7500
*SQSTM1*	0.8611	0.7963	0.8438

AUC: the area under the curve; MI: myocardial infarction; HF: heart failure.

## Data Availability

All the data generated or analyzed during this study include the available Gene Expression Omnibus (GEO) database (Accession nos. GSE59867 and GSE42955) and a published article (DOI: 10.1161/CIRCULATIONAHA.119.045158).
